# Clinical and Biochemical Determinants of Acute Kidney Injury in Acute Decompensated Heart Failure: A Prospective Observational Study From a Tertiary Care Centre in North India

**DOI:** 10.7759/cureus.112546

**Published:** 2026-07-12

**Authors:** Manan Gupta, Sunita Gupta, Harmanjeet Singh Dhillon

**Affiliations:** 1 General Medicine, Maharishi Markandeshwar University of Medical Sciences and Research, Ambala, IND

**Keywords:** acute decompensated heart failure (adhf), : acute kidney injury, cardio renal syndrome, hfref: heart failure with reduced ejection fraction, nt-probnp (n terminal-pro-b-type natriuretic peptide)

## Abstract

Background

Cardiorenal syndrome type 1 (CRS-1), manifesting as acute kidney injury (AKI) during episodes of acute decompensated heart failure (ADHF), constitutes a clinically significant complication with independent adverse effects on both short- and long-term patient outcomes. Delineating the clinical, echocardiographic, and biochemical factors predisposing to this complication is foundational to risk-guided cardiorenal management. Prospective regional data from the Indian subcontinent addressing this clinical question remain insufficient.

Materials and methods

A prospective observational investigation enrolled 100 sequential adults who met the 2021 European Society of Cardiology (ESC) criteria for ADHF and were admitted to the Intensive Cardiac Care Unit and Medical ICU at Maharishi Markandeshwar University of Medical Sciences and Research (MMIMSR), Ambala, Haryana, India. AKI was ascertained using the Kidney Disease: Improving Global Outcomes (KDIGO) 2012 serum creatinine thresholds after exclusion of competing aetiologies. Comorbid diagnoses, echocardiographic indices (Left Ventricular Ejection Fraction (LVEF), phenotypic classification), and admission biochemistry including serum sodium and N-terminal pro-B-type natriuretic peptide (NT-proBNP) were prospectively documented. Associations between different variables and AKI were evaluated using Chi-square analysis, independent-samples t-test or Mann-Whitney *U* test, unadjusted odds ratios (OR) with 95% confidence intervals, and ROC curve analysis. A two-tailed *p*-value below 0.05 denoted statistical significance.

Results

Half the enrolled cohort (50%) fulfilled criteria for AKI. The Heart Failure with Preserved Ejection Fraction (HFrEF) phenotype (LVEF ≤40%) was present in 80.0% of AKI patients relative to 68.0% of AKI-free patients (OR 1.88; 95% CI 0.73-4.82; χ² = 1.87; p = 0.171). Among comorbid conditions, only diabetes mellitus attained statistical significance (OR 4.37; 95% CI 1.89-10.08; χ² = 11.95; p < 0.001). Biochemically, AKI patients exhibited substantially higher median NT-proBNP (16,832.5 vs 6,599.5 pg/mL; p < 0.001), and lower serum sodium (135.96 ± 4.38 vs 137.98 ± 4.03 mEq/L; p = 0.018). NT-proBNP yielded the greatest discriminatory capacity (AUC 0.74; 95% CI 0.64-0.83), ahead of serum sodium (area under the curve (AUC) 0.64) and LVEF (AUC 0.60).

Conclusion

AKI is encountered in half of patients hospitalised with ADHF at a North Indian tertiary centre. Diabetes mellitus represents the predominant metabolic risk determinant, while admission NT-proBNP furnishes the strongest single-biomarker discriminatory signal. These routinely obtainable parameters constitute a clinically actionable framework for cardiorenal risk stratification at hospital admission.

## Introduction

Heart failure constitutes a major global public health challenge, affecting an estimated 37.7 million individuals, of whom 13-23 million reside in the Indian subcontinent. Emergency hospitalisation for acute decompensation accounts for a substantial share of this burden; in-hospital fatality rates span 5-15%, and nearly one in four discharged patients is re-admitted within 30 days [1,2]. Among the organ-specific sequelae accompanying ADHF, acute kidney injury (AKI) is of paramount clinical relevance, occurring in 9-62% of hospitalised patients and independently conferring heightened mortality risk, prolonged hospitalisation, elevated readmission rates, and accelerated progression toward chronic kidney disease [3,4]. The pathophysiological basis of AKI in acute decompensated heart failure (ADHF) is cardiorenal syndrome type 1 (CRS-1), in which acute haemodynamic and neurohormonal derangements from cardiac decompensation precipitate renal functional decline. Operative mechanisms include diminished cardiac output producing renal hypoperfusion, venous congestion elevating renal venous pressure with impairment of glomerular filtration, and neurohormonal activation of the renin-angiotensin-aldosterone system (RAAS) and sympathetic axis inducing afferent arteriolar vasoconstriction [5,6]. The relative prominence of each pathway differs by heart failure phenotype; systolic dysfunction (HFrEF) typically imparts a more pronounced haemodynamic perturbation than preserved ejection fraction states [7].

Prospective identification of patients vulnerable to AKI during an ADHF admission would enable targeted escalation of renal surveillance, judicious diuretic management, timely nephrology consultation, and avoidance of nephrotoxic exposures. Nonetheless, robust prospective data from the Indian subcontinent that quantify the clinical, echocardiographic, and biochemical determinants of AKI in ADHF, alongside their individual discriminatory performance, remain scarce. Most published cohort data derive from Western, East Asian, or sub-Saharan African populations whose comorbidity profiles and heart failure phenotype distributions diverge meaningfully from those encountered in North India [8]. Motivated by this evidence gap, the current study was initiated with four principal objectives: (i) to determine the prevalence of AKI among ADHF patients admitted to a North Indian tertiary facility; (ii) to identify clinical, echocardiographic, and biochemical variables associated with AKI development; (iii) to quantify the discriminatory performance of continuous predictor variables through receiver operating characteristic (ROC) analysis; and (iv) to compare in-hospital outcomes between patients with and without AKI.

## Materials and methods

Study design and setting

This prospective observational study was conducted between November 2023 and May 2026 in the Intensive Cardiac Care Unit (ICCU) and Medical Intensive Care Unit of Maharishi Markandeshwar Institute of Medical Sciences and Research (MMIMSR), Ambala, Haryana, India. Ethical approval was granted by the Institutional Ethics Committee of MMIMSR (IEC Approval No. IEC-3215), and the investigation was conducted in full conformity with the 2013 Declaration of Helsinki. Written informed consent was obtained from all participants or their legally authorised representatives in their preferred regional language before study entry.

Participant selection

Consecutive adults aged ≥18 years admitted with a confirmed ADHF diagnosis were enrolled. Diagnostic confirmation adhered to the 2021 European Society of Cardiology (ESC) Heart Failure Guidelines, requiring the presence of characteristic symptoms (dyspnoea, ankle oedema, fatigue)and signs (elevated jugular venous pressure, pulmonary crepitations or rhonchi, peripheraloedema) attributable to underlying structural or functional cardiac pathology [9]. Exclusion criteria encompassed: acute coronary syndrome (ST-segment elevation myocardial infarction (STEMI) or non-ST-elevation myocardial infarction (NSTEMI)), cardiac tamponade, malignancy, pulmonary thromboembolism, sepsis, established chronic kidney disease (CKD), contrast-induced nephropathy, and end-stage renal disease (ESRD). Exclusion of these conditions was implemented to ensure that any observed renal deterioration reflected genuine CRS-1 rather than an alternative aetiology.

Definitions

AKI was diagnosed per KDIGO 2012 serum creatinine criteria as: (a) an absolute serum creatinine elevation of ≥0.3 mg/dL within a 48-hour window, or (b) a proportional increase to ≥1.5 times the previously established baseline within seven days [10]. Given universal loop diuretic exposure in this population, urine output-based Kidney Disease: Improving Global Outcomes (KDIGO) criteria were withheld to avoid confounding by pharmacological diuresis. Echocardiographic phenotyping by biplane Simpson's method classified patients as HFrEF (LVEF ≤40%), HFmrEF (LVEF 41-49%), or HFpEF (LVEF ≥50%) per ESC 2021 guidance [9].

Data collection

A pre-specified case record form captured: demographic information (age, sex); presenting symptomatology and clinical signs; comorbid diagnoses (hypertension, diabetes mellitus, coronary artery disease, arrhythmia, cardiogenic shock); 12-lead ECG findings; 2D echocardiographic data including LVEF by biplane Simpson's method, chamber dimensions, wall motion abnormalities, and valvular pathology; and admission laboratory investigations including complete blood count, renal function tests, serum electrolytes with sodium, and NT-proBNP assayed by validated electrochemiluminescence immunoassay. In-hospital endpoints, including mortality, length of admission, ventilator use, and inotropic requirements, were recorded prospectively for all participants.

Statistical analysis

Analyses were conducted using IBM SPSS Statistics version 26.0 (IBM Corp., Armonk, NY). Normally distributed continuous variables are presented as mean ± standard deviation (SD); non-normally distributed variables as median with interquartile range (IQR). Categorical data appear as counts and percentages. Between-group comparisons utilised the independent samples t-test (normally distributed continuous variables), the Mann-Whitney U test (non-normally distributed continuous variables), or Pearson’s Chi-square (χ²) test (categorical variables), with Fisher’s exact test applied where expected cell frequencies were fewer than five. Unadjusted ORs with 95% CIs were derived for categorical predictors. ROC curve analysis generated AUC values with 95% CI by the DeLong method and Youden-optimal cut-offs (J = sensitivity + specificity − 1) for continuous predictors. Statistical significance was defined at a two-tailed alpha of 0.05 (p < 0.05) for all analyses. Continuous variables are presented as mean ± SD; non-normally distributed variables as median with interquartile range (IQR). Categorical data appear as counts and percentages. Between-group comparisons utilised the independent samples t-test, Mann-Whitney U test, or Chi-square test depending on variable type and distribution. Unadjusted ORs with 95% CIs were derived for categorical predictors. ROC curve analysis generated AUC values with 95% CI by the DeLong method and Youden-optimal cut-offs (J = sensitivity + specificity − 1) for continuous predictors. Statistical significance was defined at a two-tailed alpha of 0.05 (p < 0.05) for all analyses.

## Results

Cohort overview

One hundred patients were enrolled. Mean age was 60.73 ± 13.87 years (range 28-96 years); 55% were male and 45% female. The predominant HF phenotype was HFrEF (65%), followed by HFpEF (21%) and HFmrEF (14%). Overall in-hospital mortality was 8% (n=8). Dyspnoea was universal (100%); ankle swelling and fatigue were documented in 50% and 21% of participants, respectively. Elevated jugular venous pressure was present in 73%, pulmonary crepitations in 86%, and peripheral oedema in 54%. AKI satisfying KDIGO serum creatinine criteria was identified in 50 of 100 patients (50.0%). Baseline characteristics stratified by AKI status are presented in Table 1. 

**Table 1:** Baseline demographic, clinical, and laboratory characteristics of the study cohort stratified by AKI status (n = 100) Continuous normally distributed variables compared by independent samples t-test (mean ± SD). NT-proBNP compared by Mann–Whitney U test (median [IQR]). Categorical variables compared by Pearson’s χ² test or Fisher’s exact test. Statistical significance: two-tailed p < 0.05. *Denotes significant difference (p < 0.05). — = statistical test not separately reported (subgroups of HF phenotype classification). AKI = acute kidney injury; SD = standard deviation; IQR = interquartile range; HFrEF = heart failure with reduced ejection fraction (LVEF ≤40%); HFpEF = heart failure with preserved ejection fraction (LVEF ≥50%); HFmrEF = heart failure with mildly reduced ejection fraction (LVEF 41–49%); LVEF = left ventricular ejection fraction; NT-proBNP = N-terminal pro-B-type natriuretic peptide; t = independent samples t-test statistic; U = Mann–Whitney U statistic; χ² = Pearson’s chi-square statistic; n = number of patients; mEq/L = milliequivalents per litre; pg/mL = picograms per millilitre; g/dL = grams per decilitre.

Variable	AKI (n=50)	No AKI (n=50)	Statistic	p-value
Age (years), mean ± SD	61.42 ± 13.77	60.04 ± 14.31	t = 0.50	0.621
Male sex, n (%)	28 (56.0%)	27 (54.0%)	χ² = 0.04	0.842
HFrEF (LVEF ≤40%), n (%)	40 (80.0%)	34 (68.0%)	χ² = 1.87	0.171
HFpEF (LVEF ≥50%), n (%)	6 (12.0%)	12 (24.0%)	—	—
HFmrEF (LVEF 41–49%), n (%)	4 (8.0%)	4 (8.0%)	—	—
Serum sodium (mEq/L), mean ± SD	135.96 ± 4.38	137.98 ± 4.03	t = −2.40	0.018*
LVEF (%), mean ± SD	30.66 ± 12.36	33.48 ± 15.84	t = −0.99	0.323
NT-proBNP (pg/mL), median (IQR)	16,832.5 (9,226–24,795)	6,599.5 (2,842–11,587)	U = 577	<0.001*
Haemoglobin (g/dL), mean ± SD	10.89 ± 2.76	11.50 ± 2.43	t = −1.18	0.241
In-hospital mortality, n (%)	6 (12.0%)	2 (4.0%)	Fisher’s exact	0.269

Comorbidity associations with AKI

Of the five evaluated comorbidities, only diabetes mellitus demonstrated a statistically significant relationship with AKI occurrence. Glycaemic dysregulation was documented in 29 of 50 AKI patients (58.0%) versus 12 of 50 non-AKI patients (24.0%), yielding an unadjusted OR of 4.37 (95% CI 1.89-10.08; χ² = 11.95; p < 0.001). Systemic hypertension, coronary artery disease, arrhythmia, and cardiogenic shock displayed no significant differential distribution between groups (all p > 0.05). The complete comorbidity analysis appears in Table 2.

**Table 2:** Association of comorbid conditions with AKI in ADHF - Chi-square analysis and unadjusted odds ratios Between-group differences assessed using Pearson’s Chi-square (χ²) test. Unadjusted ORs with 95% CIs calculated for each comorbidity. Statistical significance: two-tailed p < 0.05. *Denotes statistically significant association (p < 0.05). AKI = acute kidney injury; ADHF = acute decompensated heart failure; OR = odds ratio; CI = confidence interval; χ² = Pearson’s Chi-square statistic; n = number of patients; % = percentage of group total.

Comorbidity	AKI n (%)	No AKI n (%)	χ²	p-value	OR (95% CI)
Diabetes mellitus	29 (58.0%)	12 (24.0%)	11.95	<0.001*	4.37 (1.89–10.08)
Hypertension	23 (46.0%)	21 (42.0%)	0.16	0.687	1.18 (0.53–2.64)
Coronary artery disease	24 (48.0%)	20 (40.0%)	0.65	0.420	1.40 (0.62–3.14)
Arrhythmia	13 (26.0%)	14 (28.0%)	0.05	0.822	0.90 (0.37–2.17)
Cardiogenic shock	4 (8.0%)	9 (18.0%)	2.21	0.137	0.41 (0.12–1.43)

Laboratory and echocardiographic predictors

Three quantitative parameters distinguished AKI from non-AKI patients (Table 2). Admission serum sodium was depressed in patients who developed AKI (135.96 ± 4.38 vs 137.98 ± 4.03 mEq/L; t = −2.40; p = 0.018), consistent with heightened neurohormonal activation. Median NT-proBNP was approximately 2.55-fold greater in the AKI group (16,832.5 [IQR 9,226-24,795] vs 6,599.5 [IQR 2,842-11,587] pg/mL; U = 577; p < 0.001), indicating more severe haemodynamic decompensation. Haemoglobin, total leucocyte count, and platelet count were comparable between groups.

**Table 3:** Comparison of continuous laboratory and echocardiographic parameters between AKI and non-AKI groups Normally distributed variables compared by independent samples t-test (mean ± SD). NT-proBNP compared by Mann–Whitney U test (median [IQR]). Statistical significance: two-tailed p < 0.05. *Denotes significant difference (p < 0.05). †NT-proBNP was non-normally distributed; the U statistic (smaller complementary value) is reported. AKI = acute kidney injury; SD = standard deviation; IQR = interquartile range; LVEF = left ventricular ejection fraction; NT-proBNP = N-terminal pro-B-type natriuretic peptide; TLC = total leucocyte count; t = independent samples t-test statistic; U = Mann–Whitney U statistic; mEq/L = milliequivalents per litre; pg/mL = picograms per millilitre; g/dL = grams per decilitre; ×10³/µL = cells × 10³ per microlitre.

Parameter	AKI (n=50)	No AKI (n=50)	Test Statistic	p-value
Serum sodium (mEq/L), mean ± SD	135.96 ± 4.38	137.98 ± 4.03	t = −2.40	0.018*
LVEF (%), mean ± SD	30.66 ± 12.36	33.48 ± 15.84	t = −0.99	0.323
NT-proBNP (pg/mL), median (IQR)†	16,832.5 (9,226–24,795)	6,599.5 (2,842–11,587)	U = 577	<0.001*
Haemoglobin (g/dL), mean ± SD	10.89 ± 2.76	11.50 ± 2.43	t = −1.18	0.241
TLC (×10³/µL), mean ± SD	9.38 ± 2.99	9.85 ± 2.84	t = −0.79	0.430
Platelet count (×10³/µL), mean ± SD	194.74 ± 87.29	202.54 ± 89.16	t = −0.44	0.659

ROC analysis of continuous predictors

NT-proBNP demonstrated the strongest predictive discrimination (AUC 0.74; 95% CI 0.64-0.83; p<0.001) at an optimal admission threshold of 10,200 pg/mL (sensitivity 66%, specificity 76%, positive predictive value [PPV] 73%, negative predictive value [NPV] 70%). Serum sodium yielded an AUC of 0.64 (95% CI 0.52-0.74; p=0.018) at a Youden-optimal cut-off of ≤136.8 mEq/L (sensitivity 66%, specificity 78%). LVEF exhibited the lowest discriminatory capacity (AUC 0.60; 95% CI 0.50-0.70; p=0.033) at a threshold of ≤32%. Complete ROC metrics for all three predictors are presented in Table 4.

**Table 4:** ROC curve analysis - discriminatory performance of continuous predictors for AKI in ADHF AUC values with 95% CI computed by the DeLong method. Optimal cut-offs identified by Youden index (J = sensitivity + specificity − 1). Statistical significance: two-tailed p < 0.05. *Denotes AUC significantly greater than 0.5 (p < 0.05). AUC = area under the receiver operating characteristic curve; CI = confidence interval; Sens. = sensitivity; Spec. = specificity; PPV = positive predictive value; NPV = negative predictive value; NT-proBNP = N-terminal pro-B-type natriuretic peptide; LVEF = left ventricular ejection fraction; pg/mL = picograms per millilitre; mEq/L = milliequivalents per litre; AKI = acute kidney injury; ADHF = acute decompensated heart failure.

Predictor	AUC (95% CI)	p-value	Cut-off	Sens.	Spec.	PPV	NPV
NT-proBNP (pg/mL)	0.74 (0.64–0.83)	<0.001*	≤10,200	66%	76%	73%	70%
Serum sodium (mEq/L)	0.64 (0.52–0.74)	0.018*	≤136.8	66%	78%	68%	76%
LVEF (%)	0.60 (0.50–0.70)	0.033*	≤32%	64%	58%	58%	64%

In-hospital outcomes

In-hospital mortality was numerically greater in AKI patients (12.0% [n=6] vs 4.0% [n=2]; unadjusted OR 3.27; 95% CI 0.60-17.80; Fisher's exact p=0.269). Although this represents a clinically meaningful threefold trend, it did not attain statistical significance. Median hospital stay was 5 days (IQR 3-8.75) vs 4.5 days (IQR 3-7); Mann-Whitney U=1281; p=0.832. Ventilator and inotropic requirements were numerically greater in the AKI group (χ²=0.233; p=0.629 and χ²=0.585; p=0.444, respectively), though neither reached significance. No patient in either group required renal replacement therapy during the index admission. Outcome data are consolidated in Table 5.

**Table 5:** In-hospital outcomes stratified by AKI status Categorical outcomes compared by Pearson's Chi-square (χ²) test or Fisher's exact test (expected cell frequency<5). Hospital length of stay compared by Mann–Whitney U test (U=1281; p=0.832), reported as median (IQR). Statistical significance: two-tailed p<0.05. No outcome reached statistical significance. AKI=acute kidney injury; n=number of patients; IQR=interquartile range; χ²=Pearson's chi-square statistic; U=Mann–Whitney U statistic; Fisher's exact=Fisher's exact test (expected cell frequency<5); %=percentage of group total; —=not applicable (no patient in either group required renal replacement therapy during the index admission).

Outcome	AKI (n=50)	No AKI (n=50)	Test	p-value
In-hospital mortality, n (%)	6 (12.0%)	2 (4.0%)	Fisher's exact	0.269
Median hospital stay, days (IQR)	5 (3–8.75)	4.5 (3–7)	Mann–Whitney U = 1281	0.832
Ventilator support required, n (%)	12 (24.0%)	10 (20.0%)	χ² = 0.233	0.629
Inotropic support required, n (%)	11 (22.0%)	8 (16.0%)	χ² = 0.585	0.444
Renal replacement therapy required, n (%)	0 (0%)	0 (0%)	—	—

## Discussion

The principal finding of this prospective study is that half of ADHF patients presenting to a North Indian tertiary care facility developed AKI satisfying KDIGO serum creatinine criteria. This 50% prevalence is concordant with the upper range of published global estimates (9.6-62%) and closely mirrors rates from comparable Indian investigations, suggesting that ADHF severity at tertiary presentation in the Indian subcontinent is systematically more advanced than in community-based populations [3,8,11].

Within heart failure (HF) phenotype strata, HFrEF emerged as the pre-eminent structural substrate for AKI, present in 80% of AKI patients versus 68% of those without AKI (OR 1.88; 95% CI 0.73-4.82; p=0.171). The phenotype-stratified CRS-1 gradient, 54.1%, 50.0% and 33.3% across HFrEF, HFmrEF, and HFpEF, respectively, reflects a mechanistically coherent pattern. HFrEF exerts a dual haemodynamic insult on renal function: forward failure diminishes renal perfusion pressure, while backward failure raises renal venous pressure through elevated left ventricular filling pressures. Mullens et al. (2009), employing invasive haemodynamic monitoring in 145 advanced ADHF patients, established that elevated central venous pressure was the principal determinant of declining renal function, outranking cardiac index [6]. Doshi et al. (2020), analysing over 2 million ADHF hospitalisations in the United States National Inpatient Sample, demonstrated that in-hospital mortality was significantly higher among HFrEF patients with AKI (adjusted OR 3.85) compared to those with HFpEF (adjusted OR 3.21), confirming the phenotype-specific vulnerability to cardiorenal injury [12]. The epidemiology, mechanisms, and clinical significance of heart failure-associated kidney dysfunction, including the mechanistic basis for HFrEF's greater cardiorenal risk, have been comprehensively reviewed by Schefold et al. (2016) [13]. The non-significant OR of 1.88 in our cohort likely reflects the high baseline prevalence of HFrEF across both groups in this ICCU-based tertiary population.

NT-proBNP achieved the highest discriminatory power (AUC 0.74), outperforming serum sodium (AUC 0.64) and LVEF (AUC 0.60) as standalone predictors. This hierarchy is mechanistically coherent: NT-proBNP integrates real-time left ventricular filling pressure, myocardial wall stress, and neurohormonal activation, the same forces that simultaneously drive venous congestion, RAAS stimulation, and renal hypoperfusion in CRS-1 [5]. Serum sodium, reflecting downstream neurohormonal consequences such as non-osmotic AVP release and RAAS-mediated water retention, is a more distal and indirect surrogate, accounting for its lower AUC. LVEF captures structural impairment rather than current decompensation severity, consistent with its modest AUC of 0.60. The significant inverse correlation between serum sodium and NT-proBNP (r = −0.38; p<0.001) and positive correlation between serum sodium and LVEF (r = +0.31; p = 0.002) confirm that all three parameters represent interconnected dimensions of a shared cardiorenal pathophysiological cascade.

Among comorbidities, diabetes mellitus was the sole condition achieving significance (OR 4.37; χ² = 11.95; p < 0.001), corroborating the distinctive renal vulnerability of diabetic patients confronting superimposed cardiorenal haemodynamic stress. Diabetic nephropathy engenders glomerulosclerosis, afferent arteriolar hyalinosis, and impaired renal autoregulation, structural changes that deplete renal functional reserve and render glomerular filtration critically dependent on systemic perfusion pressure. When ADHF then imposes further reductions in cardiac output and elevations in venous pressure, the diabetic kidney, lacking compensatory reserve, sustains disproportionate injury. This conclusion is independently corroborated by Mezgebu et al. (2024) [14], who identified DM as the strongest predictor (adjusted OR 9.55), and by Turkistani et al. (2025) [15], who confirmed the association (OR 2.26; p<0.001).

The numerically higher but statistically non-significant mortality in the AKI group (12% vs 4%; OR 3.27; p=0.269) is congruent with published global data. Thanapongsatorn et al. (2024) reported in-hospital mortality of 10.4% in true CRS-1 versus 4.3% in non-AKI ADHF [16], closely paralleling our figures. The lack of significance reflects the constrained statistical power of a 100-patient cohort for mortality as a primary endpoint; a post hoc calculation indicates that demonstrating the observed 8-percentage-point difference at 80% power and α=0.05 would require approximately 190 patients. The demographic and clinical profile of our cohort, mean age 60.73 years, HFrEF predominance, and high comorbidity burden, is consistent with comparable Indian ADHF series, reflecting the well-established earlier onset of heart failure in the Indian subcontinent relative to Western populations [17]. The haemodynamic mechanisms underpinning the adverse outcomes associated with AKI in ADHF, elevated central venous pressure driving renal congestion and progressive cardiorenal neurohormonal activation, have been extensively characterised by Damman et al. (2009) [18] and Metra et al. (2012) [19]. Gheorghiade et al. (2007) documented a graded relationship between lower admission serum sodium and higher mortality in hospitalised heart failure patients, further underscoring the prognostic significance of neurohormonal dysregulation, as reflected by hyponatraemia, in this population [20].

Limitations of this investigation include: the single-centre design, which constrains generalisability; non-application of urine output-based KDIGO criteria; and absence of post-discharge renal outcome follow-up. A key methodological limitation is the exclusive reliance on unadjusted univariate analyses. Multivariable logistic regression, the appropriate method for identifying independent predictors of the binary AKI outcome after simultaneous adjustment for potential confounders, was precluded by the available sample size; applying the standard criterion of a minimum of 10 outcome events per predictor variable, our cohort of 50 AKI events could reliably accommodate no more than five simultaneous predictors, making a fully adjusted model statistically untenable. This constraint has specific implications for individual variables: the counterintuitive distribution of cardiogenic shock (more prevalent in the non-AKI group) likely reflects confounding by treatment intensity that multivariate adjustment could have formally addressed; the non-significant association of LVEF (p=0.323) may partly reflect collinearity with NT-proBNP (r=+0.31; p=0.002); and whether NT-proBNP retains independent discriminatory value after simultaneous adjustment for diabetes mellitus, tachyarrhythmias, and HFrEF phenotype cannot be determined from the current data. Multivariable linear regression, applicable to exploring independent determinants of NT-proBNP and LVEF, was similarly constrained; the intercorrelation between these two variables (r=+0.31; p=0.002), the sparse cell counts for cardiogenic shock (n=13 total) and tachyarrhythmias (n=27 total), and the non-normal distribution of NT-proBNP requiring logarithmic transformation collectively rendered a stable multivariable linear regression model analytically untenable in this cohort. All reported odds ratios therefore represent unadjusted estimates and no variable should be interpreted as an independent predictor of AKI on the basis of these analyses alone. The ROC-derived thresholds for NT-proBNP (≤10,200 pg/mL) and serum sodium (≤136.8 mEq/L) were derived from this single cohort and require external validation before clinical implementation. Multicentre prospective studies incorporating multivariable logistic and linear regression with simultaneous predictor adjustment would enable formal identification of independent AKI determinants and construction of a validated cardiorenal risk score for ADHF.

## Conclusions

This prospective study establishes that half of ADHF patients admitted to a North Indian tertiary care facility meet KDIGO criteria for AKI. Among comorbidities, diabetes mellitus (OR 4.37; p < 0.001) is the exclusive condition with independent predictive significance. Elevated admission NT-proBNP (AUC 0.74; optimal cut-off 10,200 pg/mL) consolidates haemodynamic severity into the strongest single quantifiable biomarker. Serum sodium (AUC 0.64) provides complementary discriminatory information. All parameters are obtainable at hospital admission, supporting their integration into a practical bedside framework for prospective AKI risk stratification in ADHF. These findings contribute substantive Indian subcontinent-specific evidence to the growing global literature on cardiorenal interactions in heart failure.
